# Chondrocytes From Osteoarthritic and Chondrocalcinosis Cartilage Represent Different Phenotypes

**DOI:** 10.3389/fcell.2021.622287

**Published:** 2021-04-26

**Authors:** Franziska Meyer, Annalena Dittmann, Uwe Kornak, Maria Herbster, Thomas Pap, Christoph H. Lohmann, Jessica Bertrand

**Affiliations:** ^1^Department of Orthopaedic Surgery, Otto-von-Guericke University Magdeburg, Magdeburg, Germany; ^2^Institut für Humangenetik, Universitätsmedizin Göttingen, Göttingen, Germany; ^3^Institute of Experimental Musculoskeletal Medicine, University Hospital Muenster, Münster, Germany

**Keywords:** calcium pyrophosphate dihydrate, osteoarthritis, cartilage, senescence, chondrocyte, calcification, chondrocalcinosis senescence in chondrocalcinosis 2

## Abstract

Basic calcium phosphate (BCP)-based calcification of cartilage is a common finding during osteoarthritis (OA) and is directly linked to the severity of the disease and hypertrophic differentiation of chondrocytes. Chondrocalcinosis (CC) is associated with calcium pyrophosphate dihydrate (CPPD) deposition disease in the joint inducing OA-like symptoms. There is only little knowledge about the effect of CPPD crystals on chondrocytes and the signaling pathways involved in their generation. The aim of this study was to investigate the chondrocyte phenotype in CC cartilage and the effect of CPPD crystals on chondrocytes. Cartilage samples of patients with CC, patients with severe OA, and healthy donors were included in this study. The presence of CC was evaluated using standard X-ray pictures, as well as von Kossa staining of cartilage sections. OA severity was evaluated using the Chambers Score on cartilage sections, as well as the radiological Kellgren–Lawrence Score. Patients with radiologically detectable CC presented calcification mainly on the cartilage surface, whereas OA patients showed calcification mainly in the pericellular matrix of hypertrophic chondrocytes. OA cartilage exhibited increased levels of collagen X and matrix metalloproteinase 13 (MMP13) compared with CC and healthy cartilage. This observation was confirmed by qRT-PCR using cartilage samples. No relevant influence of CPPD crystals on hypertrophic marker genes was observed *in vitro*, whereas BCP crystals significantly induced hypertrophic differentiation of chondrocytes. Interestingly, we observed an increased expression of p16 and p21 in cartilage samples of CC patients compared with OA patients and healthy controls, indicating cellular senescence. To investigate whether CPPD crystals were sufficient to induce senescence, we incubated chondrocytes with BCP and CPPD crystals and quantified senescence using β-gal staining. No significant difference was observed for the staining, but an increase of p16 expression was observed after 10 days of culture. Primary chondrocytes from CC patients produced CPPD crystals in culture. This phenotype was stabilized by mitomycin C-induced senescence. Healthy and OA chondrocytes did not exhibit this phenotype. BCP and CPPD crystals seem to be associated with two different chondrocyte phenotypes. Whereas BCP deposition is associated with chondrocyte hypertrophy, CPPD deposition is associated with cellular senescence.

## Introduction

The calcification of collagenous matrix is a physiological process. Two types of calcium crystals, basic calcium phosphate (BCP) crystals and calcium pyrophosphate dihydrate (CPPD) crystals, have been described to be present in cartilage (Fuerst et al., 2009a). CPPD crystals are rhomboid shaped, 1–20 μm in size, and birefringent in polarized light (Schumacher, 1996). BCP crystals, however, are only about 1 nm in size and not birefringent (Dieppe et al., 1976).

Chondrocalcinosis (CC) can be associated with severe inflammation and massive joint destruction (Fuerst et al., 2011). The knee joint, wrists, and symphysis are frequently affected. CC affected joints can also be asymptomatic for a long time. The prevalence of CC is about 7% (United Kingdom) of the population, with a strong association with age. The prevalence rises by 3.7% in the age group 55–59 and by 17.5% in the 80–84 age group (Neame et al., 2004). CC has been described to occur bilaterally in the knee joints, with the lateral compartment and especially the meniscus being more frequently affected than the medial compartment (Neame et al., 2004). In general, CC is characterized by a deposition of CPPD crystals in the joint tissues and thus differs from osteoarthritis (OA), in which mainly BCP crystals are formed predominantly in the articular cartilage (Bjelle and Sundstrom, 1975; Fuerst et al., 2009b). The cause of CC can be sporadic or familial or associated with various metabolic abnormalities, such as hypermagnesemia and hemochromatosis (Cheng and Pritzker, 1988; Richette et al., 2005; Cimbek et al., 2015). The genetic triggers of CC are mainly mutations in the progressive ankylosis (ANK) gene and the corresponding promoter resulting in an increased expression of ANK, which promotes the formation of CPPD crystals (Zaka and Williams, 2005; Abhishek and Doherty, 2011; Abhishek et al., 2014). Furthermore, a correlation with CC and an increased activation of ectonucleotide pyrophosphatase/phosphodiesterase (NPP1) has been suggested (Derfus et al., 1996). Thereby, the pyrophosphate pathway has been described to play a critical role in the regulation of CPPD crystal deposition (Karpouzas and Terkeltaub, 1999).

The relationship between CC and OA is not clearly described in the literature. Some studies suggest a link between both conditions (Abhishek et al., 2016; Karimzadeh et al., 2017), while other studies do not describe a direct correlation between CC and OA (Fuerst et al., 2009a; Misra et al., 2015). Some studies show that CPPD crystals are detectable in about 20% of patients with OA (Fuerst et al., 2009a, b). The mechanistic link between CPPD crystal deposition and cartilage degradation in CC is not clearly described. CPPD crystals have also been shown to induce inflammatory signaling pathways in chondrocytes *in vitro* (Campillo-Gimenez et al., 2018). However, detailed mechanisms for CPPD crystal deposition in CC have not been described until now. Furthermore, the detailed effects of CPPD crystals on the joint tissues have not been investigated in detail.

Basic calcium phosphate crystal deposition has been attributed to hypertrophic differentiation of chondrocytes during OA (Fuerst et al., 2009a). Furthermore, BCP crystals have been implicated in various signaling pathways including the inflammatory signaling pathways and canonical WNT signaling (Ea et al., 2013; Nasi et al., 2016, 2017; Bertrand et al., 2020). Besides hypertrophic differentiation of chondrocytes, also apoptosis and senescence have been associated with the OA chondrocyte phenotype (McCulloch et al., 2017). Chondrocyte senescence has been published to be a hallmark of human OA cartilage (Martin et al., 2004a, b; Philipot et al., 2014). Cellular senescence is characterized by a blockade of the cell cycle by increased expression of p16 and p21, apoptosis resistance, and a characteristic secretory phenotype comprising a multitude of inflammatory cytokines (Campisi, 2007). A variety of stressors have been shown to induce senescence including genotoxic stress, e.g., by oxidative DNA damage or by shortening of telomeres after multiple cell divisions. Interestingly, in a mechanically induced OA mouse model, an accumulation of senescent cells was found in articular cartilage. The administration of “senolytic” substances, which lead to apoptosis of senescent cells, reduced cartilage damage (Jeon et al., 2017).

As it is unclear which chondrocyte phenotype is associated with CC, this study aims to investigate the chondrocyte phenotype in CC cartilage as well as the effect of CPPD crystals on chondrocytes.

## Materials and Methods

### Human Cartilage Samples

Human OA articular cartilage was obtained from patients undergoing joint replacement for knee OA after they gave written consent. Ethical approval for this study was given by the Institutional Review Board (IRB) of the Medical School, Otto-von-Guericke University, Magdeburg (IRB No. 28/20). Healthy cartilage samples were taken from the Department of Forensic Medicine during autopsies of young patients without macroscopic signs of OA or joint trauma (IRB No. 23/16). CC patients were identified using the X-ray image. Von Kossa staining of cartilage sections was used to confirm the presence of CC. OA patients were discriminated from CC patients by no visible calcification between femur and tibia in the X-ray, as well as using the von Kossa staining of cartilage sections. OA patients and CC patients are age matched; healthy controls are of younger age. Full thickness samples were dissected from most loaded areas of articular cartilage in the medial compartment of tibial plateau.

### Sample Preparation and Histological Staining

Cartilage samples were fixed in freshly prepared 4% paraformaldehyde (PFA) in phosphate-buffered saline (PBS; pH 7.4) at 4°C for 24 h. The samples were dehydrated through a graded series of ethanol solutions and embedded in paraffin. Sections (5 μm) were cut and deparaffinized and histochemically stained. Osteoarthritic changes were evaluated by staining with Safranin-Orange. Cartilage scoring was performed as described using the OARSI Scoring (Pritzker et al., 2006). Von Kossa stainings of human cartilage samples were performed to assess the location and quantity of calcification.

### Immunofluorescence Stainings

Paraffin cartilage sections were rehydrated. For aggrecan (1:500, LS Bio LS-A1561) and Col X (1:300, Abcam, #ab58632), antigen retrieval was performed with trypsin. For matrix metalloproteinase 13 (MMP13) (1:300, Abcam, #ab39012), antigen retrieval was performed with citrate buffer at pH 6. For p16 (1:70, Abcam, #ab51243), antigen retrieval was performed with citrate buffer at pH 9. Free epitopes were blocked with 4% bovine serum albumin (BSA) in PBS for 1 h at room temperature (RT). Alexa Fluor 555 (Thermo Scientific) was applied as a secondary antibody. Sections were fixed with Roti-Mount FluorCare DAPI (Roth). Control IgG stainings were performed for each antibody staining and served as an internal control for antibody specificity.

### Isolation and Culture of Chondrocytes

Primary chondrocytes were isolated from articular cartilage of patients or neonatal murine knee joints. The cartilage was removed from the femoral and tibial condyle and cut into small pieces. Cartilage pieces were incubated for 30 min with Dulbecco’s modified Eagle’s medium (DMEM) containing 1 mg/ml of Pronase (Sigma). Afterward, the medium was removed and replaced overnight with DMEM (Sigma) containing with 1 mg/ml of collagenase D (Worthington-Bio). On the following day, the cells were filtered and washed with PBS and cultured in cell culture flask.

### Induction of Cellular Senescence

Chondrocytes were seeded at a density of 2 ^∗^ 10^5^ cells/well in 6-well plates. On the two following days, the cells were treated with 200 nM of mitomycin C (Sigma-Aldrich) or the similar amount of dimethyl sulfoxide (DMSO) as a control for 24 h each. Cells were cultivated for three more days and then fixed in 4% PFA for further analyses.

### Quantification of Calcium Pyrophosphate Dihydrate Crystal Deposition

Chondrocytes were cultured for 1, 5, and 10 days in glass-bottomed wells. The cells were fixed in freshly prepared 4% PFA in PBS at room temperature for 30 min. The nuclei were stained using Roti-Mount FluorCare DAPI (Roth) for 30 min at room temperature. After they were washed in PBS, microscopic pictures were taken using polarized light at 400 × magnification. The number of crystals per DAPI-positive cell were counted using ImageJ.

### Scanning Electron Microscopy With Energy-Dispersive Spectroscopy Analyses

Chondrocyte cultures were imaged and analyzed using scanning electron microscopy (SEM) (FEI Scios DualBeam equipped with an EDAX EDS system, Thermo Fisher, United States). First, samples were sputtered with a thin gold layer (<10 nm) in order to ensure electrical conductivity. SEM investigations were executed using an acceleration voltage of 10 kV. Imaging was performed using secondary electrons, and chemical analyses were performed by energy-dispersive X-ray (EDX) spectroscopy (EDS). Regions of interest (ROIs) were identified using imaging mode at lower nominal magnifications (250×). Chemical analyses of essential ROIs were performed as EDS spot analysis.

### RNA Extraction, cDNA Synthesis, and Real-Time RT-PCR

Total RNA was extracted from cartilage explants using TRIzol reagent (Invitrogen). Total RNA of 1 μg from each sample was reverse transcribed using High-Capacity cDNA Reverse Transcription Kit (Applied Biosystems) using oligo dT primers. Quantitative PCR was performed with SYBR Green I using Applied Biosystems PRISM 7900HT (Thermo Scientific). Primer sequences are listed in Supplementary Table 1. Absolute quantification was carried out using standard curves. Target gene expression was normalized to glyceraldehyde-3-phosphate dehydrogenase (GAPDH).

### Basic Calcium Phosphate and Calcium Pyrophosphate Dihydrate Crystal Stimulation

Sterile, pyrogen-free BCP crystals were synthesized as previously described (Bertrand et al., 2020). Triclinic CPPD crystals were purchased from InvivoGen.

### Senescence-Associated β-Galactosidase Staining

Staining for senescent cells was performed on cell culture chondrocytes. The staining was performed according to the manufacturer’s instructions (Senescence β-Galactosidase Staining Kit #9860, Cell Signaling).

### Statistics

All data were presented as mean ± SEM. Data comparing two groups were analyzed by a *t*-test for statistical significance. Data with more than two groups were analyzed by a one-way analysis of variance (ANOVA) followed by a Holm–Sidak’s test as *post hoc* test in case of a statistically significant ANOVA result or a Kruskal–Wallis test in case of non-parametric data distribution with a Dunn’s *post hoc* test. A Shapiro–Wilk normality test was performed to identify parametric or non-parametric data distribution. Data analyses were performed using GraphPad Prism V.6.00 for Windows (GraphPad Software, La Jolla, CA, United States^1^). Statistical significance was determined at level of *p* ≤ 0.05.

## Results

### Chondrocalcinosis Cartilage Shows Less Severe Histological Destruction

**FIGURE 1:**
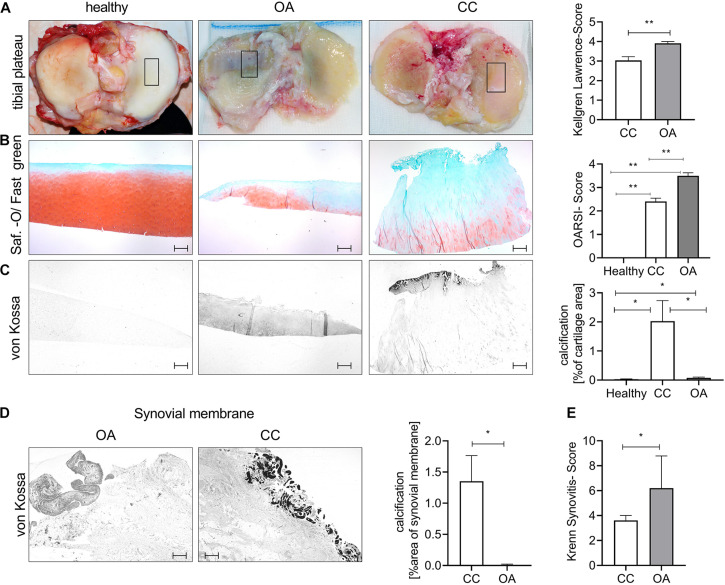
Chondrocalcinosis (CC) cartilage shows less severe histological cartilage destruction. **(A)** Representative images for the tibial plateau of healthy controls, as well as osteoarthritis (OA) and CC patients (the black square indicates the main loading area and region of sampling). The quantification of radiological OA was performed using the Kellgren–Lawrence Score [CC (3.0 ± 0.19), *N* = 27; OA (3.9 ± 0.09) *N* = 11, *t*-test *p* = 0.0075]. **(B)** Representative pictures of cartilage sections stained with Safranin-Orange (middle). Quantification of histological OA severity in the tested OA and CC cohorts [healthy: 0 (*N* = 6), CC: 2.4 ± 0.14 (*N* = 24) and OA: 3.5 ± 0.13 (*N* = 10), one-way ANOVA: *F*(2,37) = 71.69, *p* < 0.0001]. **(C)** Representative pictures of von Kossa-stained cartilage sections. Quantification of cartilage calcification from von Kossa staining [healthy: 0.03 ± 0.01 (*N* = 6, n: 1–2), CC: 2.02 ± 0.7 (*N* = 27, n: 1–2), and OA: 0.07 ± 0.02 (*N* = 11, n: 1–2), one-way ANOVA: *F*(2,76) = 4.389, *p* = 0.01]. **(D)** Representative von Kossa staining of synovial membrane from OA and CC patients, with quantification of calcification [*t*-test: CC: 1.35 ± 0.4 (*N* = 27) and OA: 0.006 ± 0.004 (*N* = 11)]. **(E)** Quantification of synovitis in CC and OA patients using the Krenn Synovitis Score [*t*-test: CC: 3.6 ± 0.4 (*N* = 27) and OA: 6.2 ± 1.2 (*N* = 5)]. The scale bar indicates 500 μm. *p ≤ 0.05, **p ≤ 0.01.

To investigate the histological cartilage changes of CC and OA cartilage, we included also healthy control cartilage samples. Furthermore, CC synovial tissue samples and OA synovial tissue samples were investigated. The age of the CC (67.04 ± 2 years) and OA (64.6 ± 4 years) patients was comparable, whereas healthy (26.25 ± 3 years) donors were younger (Supplementary Figure 1A). The radiological severity of OA was measured using the Kellgren–Lawrence Score (KL Score). We observed a significantly lower KL Score in CC patients (3.03 ± 0.2) in comparison with OA patients (3.91 ± 0.1; *p* = 0.0075) (Figure 1A). Next, we dissected cartilage from the main loading area of knee joints from patients undergoing knee joint surgery for severe OA and compared this cartilage with CC cartilage and healthy control cartilage (Figure 1B). Safranin-Orange staining with subsequent OARSI Scoring showed significantly less histological cartilage damage in CC cartilage compared with OA cartilage (*p* < 0.001). As expected, the healthy control cartilage did not show signs of cartilage degradation in the OARSI Scoring (*p* < 0.001) (Figure 1B). Interestingly, the von Kossa staining revealed less cartilage calcification in OA cartilage compared with CC cartilage (*p* = 0.049) (Figure 1C). Furthermore, OA cartilage showed more calcification than healthy cartilage (*p* = 0.035) (Figure 1C). To further investigate the source of calcification in CC, we also stained synovial membrane using von Kossa staining for the presence of calcification (Figure 1D). Again, we observed an increase in tissue calcification in the CC synovial membrane compared with OA synovial membrane (*p* = 0.05). However, this increase in calcification was not accompanied by an increase in synovitis as scored by the Krenn Synovitis Score. In contrast, we found an increased synovitis in OA synovial membrane compared with CC synovial membrane (*p* = 0.016) (Figure 1E).

### Chondrocytes in Chondrocalcinosis Cartilage Do Not Show Increased Markers of Hypertrophic Differentiation

**FIGURE 2:**
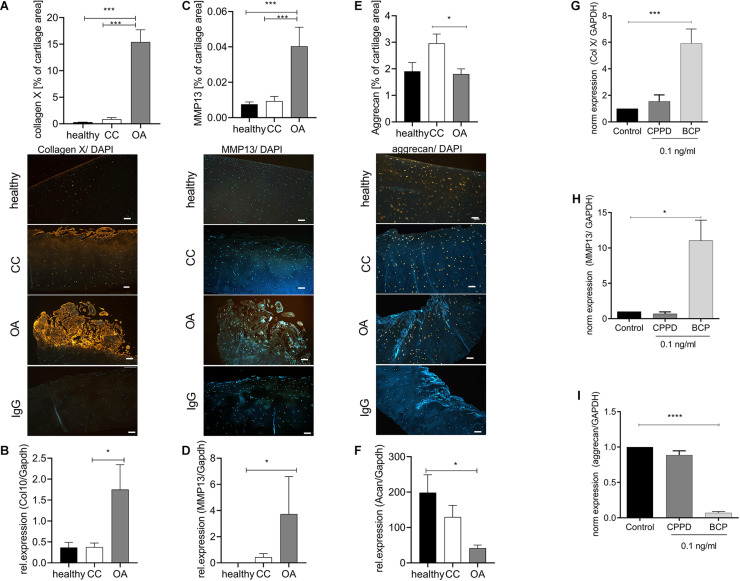
Chondrocytes in chondrocalcinosis (CC) cartilage do not show increased markers of hypertrophic differentiation. **(A)** Quantification of collagen X-stained cartilage area in healthy, CC, and osteoarthritis (OA) cartilage sections [one-way ANOVA: *F*(2,65) = 51.06, *p* < 0.0001]. Representative images of collagen X staining, as well as IgG control staining, are shown (scale bar 200 μm). **(B)** Quantitative RT-PCR for collagen X expression of cartilage specimens from healthy, OA, and CC cartilage [one-way ANOVA: *F*(2,22) = 4.12, *p* = 0.03]. **(C)** Quantification of metalloproteinase 13 (MMP13)-stained cartilage area [one-way ANOVA: *F*(2,62) = 12.31, *p* < 0.0001]. Representative images of MMP13 staining, as well as IgG control staining, are shown (scale bar 200 μm). **(D)** Quantitative RT-PCR for MMP13 expression of cartilage specimens from healthy, OA, and CC cartilage [Kruskal–Wallis test: *F*(2,22) = 10.4, *p* = 0.006]. **(E)** Quantification of aggrecan-stained cartilage area in healthy, CC, and OA cartilage sections [one-way ANOVA: *F*(2,41) = 3.796, *p* = 0.03]. Representative images of aggrecan staining, as well as IgG control staining, are shown (scale bar 200 μm). **(F)** Quantitative RT-PCR for aggrecan expression of cartilage specimens from healthy, OA, and CC cartilage [one-way ANOVA: *F*(2,12) = 5.043, *p* = 0.025]. **(G)** Quantitative RT-PCR for collagen X expression of C28 chondrocytes stimulated with 0.1 ng/ml of either calcium pyrophosphate dihydrate (CPPD) or basic calcium phosphate (BCP) crystals [one-way ANOVA: *F*(3,10) = 9.86, *N* = 3–5, *p* = 0.0025]. **(C)** Quantification of MMP13-stained cartilage area (*t*-test: *N* > 13, *p* = 0.0003). **(H)** Quantitative RT-PCR for MMP13 expression of C28 chondrocytes stimulated with 0.1 ng/ml of either CPPD or BCP crystals [one-way ANOVA: *F*(3,12) = 4.563, *N* = 3–7, *p* = 0.024]. **(I)** Quantitative RT-PCR for aggrecan expression of C28 chondrocytes stimulated with 0.1 ng/ml of either CPPD or BCP crystals [one-way ANOVA: *F*(3,12) = 216.4, *N* = 3–7, *p* < 0.0001]. *p ≤ 0.05, ***p ≤ 0.001, ****p ≤ 0.0001.

Next, we investigated whether CC cartilage also expresses increased markers of hypertrophic chondrocyte differentiation, as observed in OA cartilage. As expected, we observed an about 30-fold increase in collagen X staining, as a marker for chondrocyte hypertrophy in OA cartilage, compared with healthy and CC cartilage (Figure 2A). This observation was confirmed by five-fold increase of collagen X expression in OA cartilage specimens compared with healthy or CC cartilage (Figure 2B). Confirming the increase in hypertrophic marker gene expression in OA cartilage, we observed a six-fold increase in MMP13 staining in OA cartilage, which was not present in healthy or CC cartilage (Figure 2C). Again, this observation was confirmed by a marked increase in MMP13 expression in OA cartilage specimens and about double the expression level as compared with CC cartilage (Figure 2D). To show the influence of CC on aggrecan, as a marker of healthy chondrocytes, we stained again tissue sections. We observed significant reduction of aggrecan staining in OA cartilage compared with CC cartilage. However, there was no obvious change between healthy and OA cartilage in aggrecan staining (Figure 2E). On expression level, a significant decrease in aggrecan expression in OA cartilage samples compared with healthy was observed, whereas no changes in expression were observed compared with CC cartilage (Figure 2F). As it is known that BCP crystals are sufficient to induce upregulation hypertrophic marker gene expression of chondrocytes, we tested the effect of BCP and CPPD crystals on the respective genes.

We observed a six-fold increase of collagen X expression in BCP-stimulated chondrocytes, but not CPPD crystal-stimulated chondrocytes (Figure 2G). A similar result was achieved for MMP13, where CPPD crystals had no effect on MMP13 expression (Figure 2H). The CPPD crystals did not also influence the aggrecan expression, whereas BCP crystals induced a 16-fold downregulation of aggrecan expression (Figure 2I).

### Cellular Senescence Markers Are Increased in Chondrocalcinosis Cartilage

**FIGURE 3:**
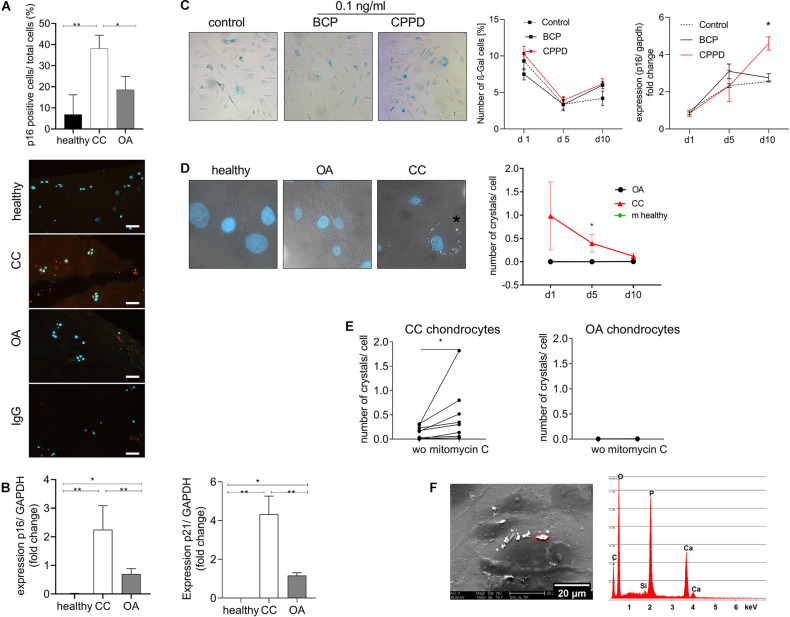
Cellular senescence markers are increased in chondrocalcinosis (CC) cartilage. **(A)** Quantification of p16-stained cartilage area in healthy, CC, and osteoarthritis (OA) cartilage sections [one-way ANOVA: *F*(2,21) = 7.064, *p* = 0.0045]. Representative images of p16 staining, as well as IgG control staining, are shown (scale bar 50 μm). **(B)** qRT-PCR for expression of senescence marker genes p16 (Kruskal–Wallis test: OA vs. CC: *p* = 0.038, CC vs. healthy: *p* = 0.0015, *N* = 5–15) and p21 (Kruskal–Wallis test: OA vs. CC: *p* = 0.05, CC vs. healthy: *p* < 0.0001, *N* = 5–15) of CC, OA, and healthy cartilage samples. **(C)** Stimulation of primary human chondrocytes with 0.1 ng/ml of basic calcium phosphate (BCP) or calcium pyrophosphate dihydrate (CPPD) crystals for up to 10 days and subsequent staining for β-Gal. Percentage of β-Gal-positive cells was counted [two-way ANOVA for time and treatment: *F*(4,30) = 1.65, *p* = 0.19]. Representative images for BCP and CPPD stimulation at day 10 are given p16 expression of chondrocytes treated with 0.1 ng/ml of BCP or CPPD crystals over the time course of 10 days [two-way ANOVA: *F*(4,15) = 3.94, *p* = 0.02]. **(D)** Culture of isolated chondrocytes from CC and OA cartilage, as well as murine neonatal chondrocytes, over a time course of 10 days. The number of crystals per cell was counted in DIC/DAPI-stained images. Representative images are given for healthy, CC, and OA chondrocytes at day 10 [two-way ANOVA: *F*(1,8) = 5.43, *p* = 0.04]. **(E)** Treatment of CC chondrocytes with mitomycin C to induce senescence. The number of crystals per cell was counted in DIC/DAPI images (CC chondrocytes: Wilcoxon-test: *p* = 0.004, *N* = 8; OA chondrocytes: *p* = 0.98, *N* = 8). **(F)** Representative electron micrograph of cultured CC chondrocytes with present crystals and energy-dispersive X-ray spectroscopy (EDS) microanalysis of a representative crystal. The microanalysis identifies the crystal as CPPD based on quantitative Ca/P ratio. *p ≤ 0.05, **p ≤ 0.01.

We did not observed an increase in hypertrophic marker genes in CC cartilage, as it is described for OA cartilage. Therefore, we investigated another chondrocyte differentiation pathway that has been linked to cartilage degeneration. Interestingly, we found a doubling of p16-positive chondrocytes in CC cartilage compared with healthy and OA cartilage (Figure 3A). To investigate a possible differentiation of the chondrocyte phenotype toward senescence in CC cartilage, we investigated the expression of p16 and p21, as senescence marker genes, in cartilage samples from OA and CC patients and healthy controls. The expression of p16 was three-fold upregulated in CC cartilage samples compared with OA cartilage (*p* = 0.003). This observation was confirmed by a three-fold increase in p21 in CC cartilage compared with OA samples (*p* = 0.05). However, an increase in p16 and p21 was also found in OA cartilage compared with healthy controls (Figure 3B). Next, we investigated whether CPPD crystals would be sufficient to induce cellular senescence *in vitro*. Therefore, we stimulated human primary human chondrocytes with 0.1 ng/ml of either BCP or CPPD crystals for up to 10 days and quantified the amount of β-galactosidase (β-Gal)-positive cells at days 1, 5, and 10. There was no change in β-Gal-positive cells for all conditions. However, we observed an increase in p16 expression after 10 days with CPPD crystal stimulation in comparison with BCP and unstimulated chondrocytes (*p* = 0.0068) (Figure 3C). Interestingly, we observed a capacity of CC chondrocytes to produce CPPD crystals without stimulation, which was not observed in either OA or murine primary chondrocytes (two-way ANOVA: *F*(1,8) = 5.43, *p* = 0.04). The phenotype of CPPD crystal production in CC chondrocytes reduced during the culturing time (Figure 3D). However, after treatment of CC chondrocytes with mitomycin C to induce senescence, the production of CPPD crystals was increased (Wilcoxon test: *p* = 0.0039) (Figure 3E). This effect was not observed in OA chondrocytes, which did not produce crystals with or without induction of senescence (Figure 3E). To verify that the observed crystals are indeed CPPD crystals, we performed SEM-EDX on chondrocyte cultures, identifying the chemical composition of the crystals. The EDX spectrum proves that the crystals produced by CC chondrocytes are CPPD crystals (Figure 3F).

## Discussion

The prevalence of CC has been associated with a female preponderance and aging, as most patients affected are over the age of 65 (Higgins, 2016). Our cohort does not show the predominance of female patients (Supplementary Figure 1A). The age of the OA and CC patients is in line with the current literature and was similar in both groups (Supplementary Figure 1B). Current literature describing CC is mainly focusing on imaging-based diagnostics of CC, but the molecular pathways underling this disease are only poorly understood. This study sheds light on the molecular pathways contributing to CPPD crystal deposition.

Chondrocalcinosis has been described to most commonly affect fibrocartilage, but also occurs in hyaline cartilage of the knee, shoulder, and hip (Abhishek and Doherty, 2011). Interestingly, we also observed calcification in the synovial membrane, giving rise to the assumption that besides chondrocytes, fibroblasts are also able to contribute to CPPD crystal deposition (Figure 1D). So far, CPPD crystals have been associated with inflammatory reactions due to the activation of the inflammasome *in vitro* (Renaudin et al., 2019). Our data, however, indicate that the inflammatory reaction of the synovial membrane in CC patients is less compared with OA (Figure 1E). These data could be interpreted in a way that either the inflammation in OA is more severe or the CC patients in this study were not in the active inflammatory phase of CC when the samples were taken.

As aging has been also associated with the CC phenotype, we investigated the presence of senescent chondrocytes in CC cartilage. Cellular senescence has been associated with OA pathology by various studies (McCulloch et al., 2017). We observed that senescent marker genes *p16* and *p21* are markedly increased in CC cartilage compared with OA cartilage. Zhou et al. (2004) reported more p16-positive chondrocytes in OA cartilage compared with age-matched normal tissue. They linked *p16* to OA pathogenesis, as *p16* knockdown resulted in more chondrocyte proliferation and matrix production (Zhou et al., 2004). A study indicated that *p21* was upregulated during the early stage of senescence. The upregulation of *p16* might be essential for maintenance of the senescent cell-cycle arrest (Stein et al., 1999). Therefore, chondrocytes in CC cartilage might be kept in a constant cell-cycle arrest, due to upregulation of both marker genes. Interestingly, we show that CC chondrocytes have the capacity to produce CPPD crystals, even when they are isolated from their pathological surrounding in the diseased joint (Figure 3D). This observation is in line with the finding that the deposition of BCP crystals is also a pathological program that started in hypertrophic chondrocytes during OA, which is kept even after isolation of chondrocytes from OA cartilage (Fuerst et al., 2009a). However, chondrocytes in CC cartilage did not express increased markers of hypertrophic differentiation (Figure 2), as they do in OA. Our data indicate that CC is associated with a senescent phenotype of chondrocytes, as both senescence markers *p16* and *p21* are upregulated in CC cartilage (Figure 3B). However, CPPD crystals themselves did only induce a minor increase in *p16* expression, but have no change in the amount of β-Gal-positive cells (Figure 3C). BCP crystals have been shown to induce hypertrophic differentiation of chondrocytes by activating the canonical Wnt signaling pathway (Bertrand et al., 2020). However, no effect of BCP crystals on chondrocyte senescence markers was detected (Figure 3C). The amount of CPPD crystals produced by CC chondrocytes reduced over time (Figure 3C). However, the amount of crystal production was significantly increased by induction of senescence using mitomycin C in CC chondrocytes (Figure 3E). Induction of senescence in OA chondrocytes was not sufficient to induce CPPD crystal deposition. This finding indicates that there is a link between senescence and CPPD crystal deposition. However, the induction of senescence alone is not sufficient to induce the phenotype as well as the stimulation with CPPD crystals. It seems that there must be metabolic changes of CC chondrocytes enabling them to produce these crystals. CC, and the deposition of CPPD crystals, is believed to be caused by an imbalance between the extracellular levels of pyrophosphate and phosphate. In theory, pyrophosphate is secreted in the synovium and adjacent tissues, where it combines with calcium to form CPPD crystals (Higgins, 2016). The present data indicate that there might be a genetic predisposition in CC patients inducing the deposition of CPPD crystals, leading to increased chondrocyte senescence.

## Conclusion

Basic calcium phosphate and calcium pyrophosphate dihydrate crystals seem to be associated with two different chondrocyte phenotypes. Whereas BCP deposition is associated with chondrocyte hypertrophy, CPPD deposition is associated with chondrocyte senescence.

## Data Availability Statement

The raw data supporting the conclusions of this article will be made available by the authors, without undue reservation.

## Ethics Statement

The studies involving human participants were reviewed and approved by the Institutional Review Board (IRB) of the Medical School, Otto-von-Guericke University, Magdeburg (IRB No: 28/20). The patients/participants provided their written informed consent to participate in this study.

## Author Contributions

FM performed most of the experiments. AD performed the chondrocyte isolation and *in vitro* CPPD deposition. UK investigated the expression of senescence marker genes in cartilage samples. MH performed the SEM-EDX experiments. TP helped in interpreting and discussing the data and writing of the manuscript. CL identified the CC and OA patients and provided the samples. JB supervised the work and wrote the manuscript. All authors contributed to the article and approved the submitted version.

## Conflict of Interest

The authors declare that the research was conducted in the absence of any commercial or financial relationships that could be construed as a potential conflict of interest.

## Footnotes

1 www.graphpad.com

## Supplementary Material

Supplementary Figure 1 Demographic data of patient cohort. **(A)** Depicts the mean age ± SEM of the patient cohort (healthy: 26.25 ± 3 years, CC: 67.04 ± 2 years and OA: 64.6 ± 4 years). **(B)** Compares the percentage of male and female patients in each cohort.

Supplementary Table 1 Human primers for quantitative real-time PCR.
