# Supplementary figures and images for: Chondrocytes From Osteoarthritic and Chondrocalcinosis Cartilage Represent Different Phenotypes
Source: Front Cell Dev Biol. 2021 Apr 26;9:622287. doi: 10.3389/fcell.2021.622287 (PMC8107373; doi:10.3389/fcell.2021.622287)

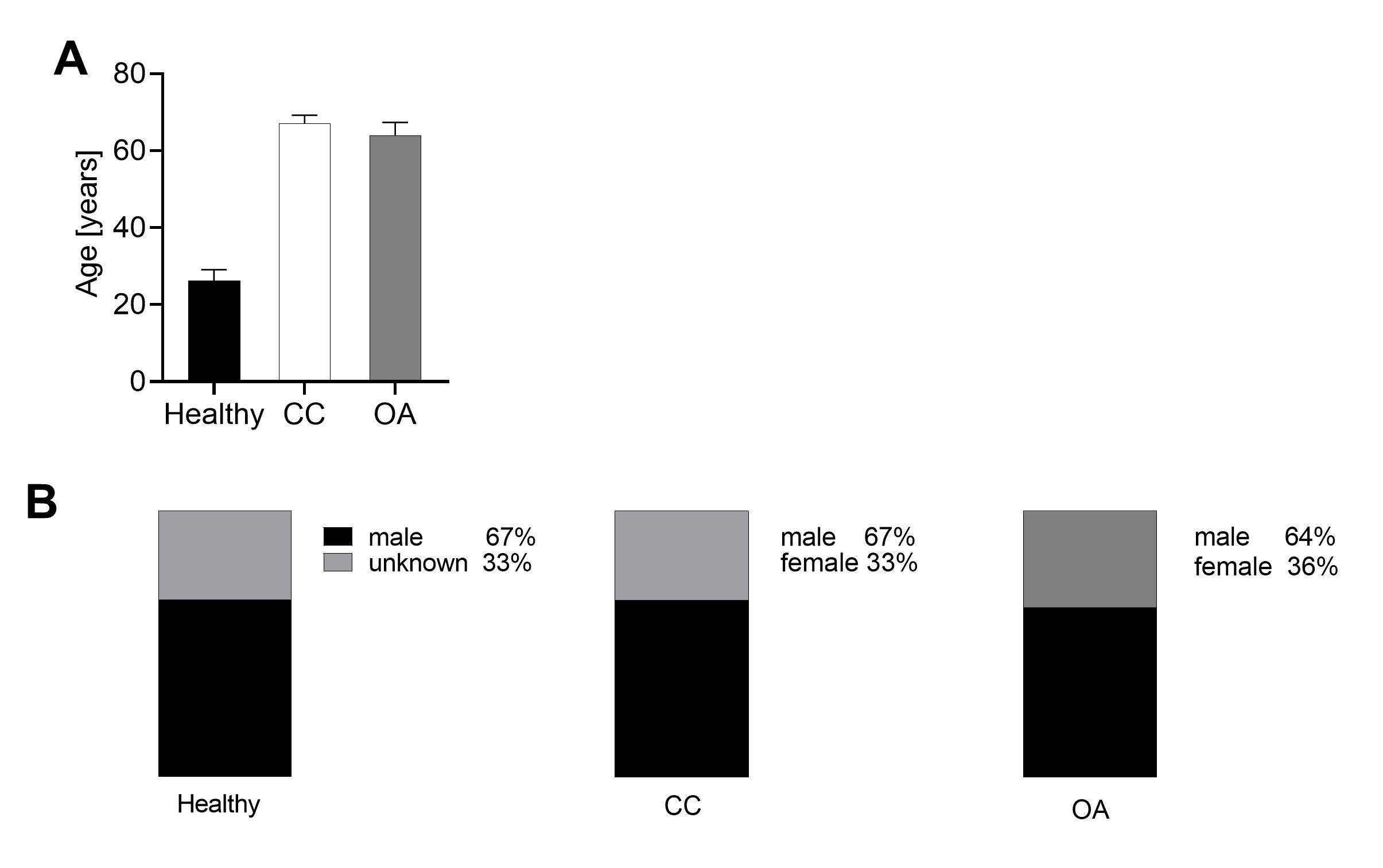

Supplement: Supplementary Figure 1 — Demographic data of patient cohort. (A) Depicts the mean age ± SEM of the patient cohort (healthy: 26.25 ± 3 years, CC: 67.04 ± 2 years and OA: 64.6 ± 4 years). (B) Compares the percentage of male and female patients in each cohort. [file Image_1.JPEG]
