# Supplementary material for: Chondrocytes From Osteoarthritic and Chondrocalcinosis Cartilage Represent Different Phenotypes
Source: Front Cell Dev Biol. 2021 Apr 26;9:622287. doi: 10.3389/fcell.2021.622287 (PMC8107373; doi:10.3389/fcell.2021.622287)
Supplement: Supplementary Table 1 — Human primers for quantitative real-time PCR. [file Table_1.DOCX]

| Gene | Sequence (in 5’ – 3’ direction) | |
| --- | --- | --- |
| aggrecan | forward  reverse | CTC CGG AAT GGA AAC GTG AAT C  CTG GTA GTC TTG GGC ATT GTT G |
| Collagen X | forward  reverse | AAT CCC TGG ACC GGC TGG AAT TTC  TTG ATG CCT GGC TGT CCT GGA ACC |
| MMP13 | forward  reverse | CTT GAC CAC TCC AAG GAC CC  GCG CCA GAA GAA TCT GTC TTT |
| NPP1 | forward  reverse | GGG TTC CTC TCC CCA CCA CAA CTA  CCA TGC ACA CAG CTC TCG CTG T |
| p16 | forward  reverse | CAA CGC ACC GAA TAG TTA CG  ACC AGC GTG TCC AGG AAG |
| p21 | forward  reverse | GGA GAC TCT CAG GGT CGA AA  CTT CCT GTG GGC GGA TTA |
| GAPDH | forward  reverse | CCC ACT CCT CCA CCT TTG AC  AGC CAA ATT CGT TGT CAT ACC AG |
